# The association between anosognosia and neuropsychiatric symptoms in neurodegenerative dementias: a narrative review

**DOI:** 10.3389/fneur.2025.1649627

**Published:** 2025-09-26

**Authors:** Chiara Gallingani, Manuela Tondelli, Patrizia Vannini, Giovanna Zamboni

**Affiliations:** ^1^Department of Biomedical, Metabolic and Neural Sciences, University of Modena and Reggio Emilia, Modena, Italy; ^2^Neurology Unit, OCB Hospital, Azienda Ospedaliero-Universitaria of Modena, Modena, Italy; ^3^Brigham and Women's Hospital, Harvard Medical School, Boston, MA, United States; ^4^Massachusetts General Hospital, Harvard Medical School, Boston, MA, United States

**Keywords:** anosognosia, unawareness, neuropsychiatric symptoms, dementia, Alzheimer’s disease, frontotemporal dementia

## Abstract

Anosognosia, or unawareness of disease, is a common clinical feature in neurodegenerative dementias. Frequently reported as an early symptom, its presence has been associated with faster dementia progression and greater cognitive impairment. Similarly, neuropsychiatric symptoms (NPS) encompass non-cognitive behavioral and psychiatric disturbances that commonly affect individuals with dementia. Both aosognosia and NPS are clinically relevant in neurodegenerative diseases due to their significant implications in disease management and caregiver burden. In this narrative review, we examined studies investigating the direct relationship between anosognosia and NPS across different neurodegenerative dementias, including Alzheimer’s Disease (AD), Frontotemporal Dementia (FTD) and *α*-synucleinopathies, such as Parkinson’s Disease (PD) and Dementia with Lewy Bodies (DLB). A total of 46 studies were identified, the majority of which focused on AD. Despite considerable heterogeneity in participant selection, assessed domains, and measures of anosognosia and NPS investigated, consistent association emerged between anosognosia and global NPS scores as well as individual symptoms. Across studies, the most common finding was a negative association between anosognosia and depression and a positive association between anosognosia and apathy. Possible underlying mechanisms and shared neuroanatomical substrates of these findings are discussed. The review provides a deepened insight into key symptoms with critical implications for dementia research, clinical management, and caregiving strategies.

## 1 Introduction

Anosognosia, also referred to as loss of insight, unawareness, or impaired self-awareness, is defined as the lack of recognition for a wide range of neurological and neuropsychological disturbances (1, 2). It is frequent in neurodegenerative dementias and can differentially affect the cognitive, behavioral, or functional impairments occurring in these diseases (3). It is estimated to affect from 20% to as many as 80% of individuals with Alzheimer’s disease (AD) dementia (2, 4, 5), with higher proportions observed when milder forms of unawareness are considered (6). Such variability is likely related to differences in assessment methods and disease severity examined across cohorts. Although precise prevalence estimates stratified by method or stage are still lacking, anosognosia is widely recognized as a relevant symptom across the whole dementia continuum. It generally worsens with dementia progression, suggesting an association with disease severity (7, 8), although some studies have reported mixed results (9, 10), or no change (11–14). Recent studies have shown that the disorder can already manifest in the predementia stages, such as Mild Cognitive Impairment (MCI) (15), or even years before symptom onset in AD mutation carriers (16). Notably, unawareness of cognitive deficits has been identified as a predictor of disease progression along the dementia continuum, from cognitively normal status to MCI and, ultimately, to AD dementia (8, 17). Accordingly, it has been proposed that reduced awareness and underreporting of cognitive deficits may serve as an early clinical marker of dementia, more specific than Subjective Cognitive Decline (SCD), supporting the usefulness of monitoring awareness in the clinical setting (18).

While anosognosia has been primarily studied in AD, it is also recognized as a significant feature of other neurodegenerative diseases. It is frequently reported in the Frontotemporal Dementia (FTD) spectrum, where it can affect up to 75% of cases and represents a hallmark feature of the behavioral variant (bvFTD) (19). Despite having been removed from the current clinical criteria (20, 21), loss of insight is again reported as a supportive feature in proposed research criteria for prodromal FTD (22). Furthermore, anosognosia has also been documented in individuals with Amyotrophic Lateral Sclerosis (ALS) exhibiting cognitive or behavioral disturbances, even in the absence of a full ALS-FTD diagnosis (23). Some studies have also reported anosognosia in *α*-synucleinopathies, including both Dementia with Lewy Bodies (DLB) and Parkinson’s Disease (PD). In DLB, unawareness of cognitive decline has been observed, though its exact prevalence remains unclear, and its presence may be influenced by AD co-pathology (24). In PD, anosognosia has been documented mostly in subjects with cognitive impairment (i.e., PD-MCI and PD dementia - PDD) and has been shown to worsen over time (25). Additionally, impaired awareness for motor symptoms has been reported in PD (26).

The presence of anosognosia in dementia has profound implications for patient care, as it can reduce adherence to treatment, impair the ability to recognize potentially harmful situations, and hinder compensatory strategies (2). These risks are even more critical in young-onset dementias, where patients are more likely to remain employed, make autonomous decisions, and care for their families (6). Overall, reduced awareness has been linked to greater caregiver burden and lower quality of life (2, 27), underscoring the need for a deeper understanding of this symptom to improve patient management.

Different methods have been used to measure anosognosia, which are commonly classified into three broad approaches (28). A first method, commonly referred to as *clinician rating*, is based on the judgment of the clinician who rates the patient’s level of awareness along an ordinal scale, following structured or unstructured interviews with the patient and the caregiver(s). The second method, *patient-informant discrepancy*, is based on the calculation of discrepancy scores of questionnaires completed by the patient and their caregiver, that asks identical questions about the patient’s functions (29). The Anosognosia Questionnaire-Dementia (AQ-D) (30) and the Everyday Cognition (ECog) scale (31) are examples of questionnaires used with this purpose. Most commonly used questionnaires also categorize different domains of anosognosia, as loss of insight may concern not only cognitive deficits but also behavioral disturbances, personality changes and loss of social cognition. Discrepancy scores can be treated as a continuum from full awareness to complete anosognosia, or cut-off values can be defined to classify subjects as aware or unaware. Clinician ratings and patient-informant discrepancies are frequently used in clinical settings to assess anosognosia in an offline way (32). The third method, *performance discrepancy*, compares the patient’s actual (objective) performance on a given neuropsychological test with their self-estimated performance. Variants of this approach have traditionally been used to assess metacognition in healthy subjects but are increasingly applied to measure online and immediate awareness in patients with dementia (33–36). At present, there is no consensus on the most accurate method to detect and characterize anosognosia, resulting in the use of different instruments across studies, sometimes also in combination. In the present review, we therefore consider the literature broadly, without focusing on a single method.

Numerous studies have investigated the relationship between anosognosia and cognitive features, consistently finding that greater unawareness is associated with poorer performance on memory and/or executive function tests (5, 29, 37, 38). In contrast, the association between anosognosia and neuropsychiatric symptoms (NPS), including potential causal links and underlying neurobiological mechanisms, remains controversial and less clearly defined. NPS encompass a wide range of non-cognitive behavioral and psychiatric manifestations (39), including but not limited to depression, apathy, affective dysregulation, impulsivity, psychotic symptoms, and, less commonly, sleep and appetite disturbances. These symptoms frequently arise in dementia, from prodromal to advanced stages (40), with prevalence rates reaching up to 97% across the dementia spectrum (41). Their expression can vary across different forms of dementia, with depression, hallucinations, and delusions being more typical of AD and DLB, whereas disinhibition and compulsions more frequent in FTD (42–44). The implications of NPS are profound, as they can lead to increased caregiver burden and reduced quality of life (45–47). They have also been associated with faster progression of cognitive decline in affected individuals and worse disease prognosis (48, 49). NPS are primarily reported by caregivers and are commonly assessed using various questionnaires, among which one of the most common is the Neuropsychiatric Inventory (NPI) (50). Understanding their expression is therefore crucial to inform both pharmacological and non-pharmacological interventions aimed at improving overall patient care and alleviating caregiver burden (51).

This narrative review aims to consolidate recent evidence on the interplay between anosognosia and NPS, identify gaps in current knowledge, and highlight potential directions for future research to inform both the scientific understanding and clinical management of these pivotal aspects of dementia.

## 2 Methods

### 2.1 Inclusion and exclusion criteria

Articles were selected based on predefined inclusion criteria: (i) studies focusing on the relationship between awareness and neuropsychiatric symptoms; (ii) studies focusing on neurodegenerative dementias, including AD, FTD, and *α*-synucleinopathies (i.e., PD, DLB); (iii) studies available in English and with full-text access. Reviews and meta-analyses were not directly included but were examined for relevant references. Additionally, relevant publications were identified and added through manual screening of the bibliographies of full-text articles. Studies were excluded if they assessed anosognosia and/or NPS in isolation without investigating their relationship, or if the study population was not sufficiently described or classified to allow reliable inferences. We also excluded animal-based studies, case reports, and clinical trials, as well as non-original publication (editorials and letters in response to previous articles), conference abstracts, and proceedings.

### 2.2 Search strategy

We performed a literature search of the MEDLINE/PubMed and Web of Science databases to identify eligible published articles from their inception to May 2, 2024. In order to capture the extent of the literature, a range of search terms were used in various combinations. The terms “awareness,” “anosognosia,” “unawareness,” and “self-insight” were used to capture the principal topic of anosognosia, while “dementia” and “neurodegenerative” to denote the group of diseases of interest. Finally, “neuropsychiatric,” “behavioral,” “behavioral,” “depression,” “apathy,” and “psychosis” were added to capture the association with NPS. We filtered the research by excluding reviews, animal studies, and articles not published in English. Retrieved articles were imported into the Rayyan Intelligent Systematic Review Tool, to remove duplicates. Articles were first screened by titles and abstracts, then the full texts of selected studies were evaluated, excluding non-eligible articles as per established criteria. Any uncertainty in the selection was discussed with the senior authors (MT, GZ, PV) until a consensus was reached.

## 3 Results

**Figure 1:**
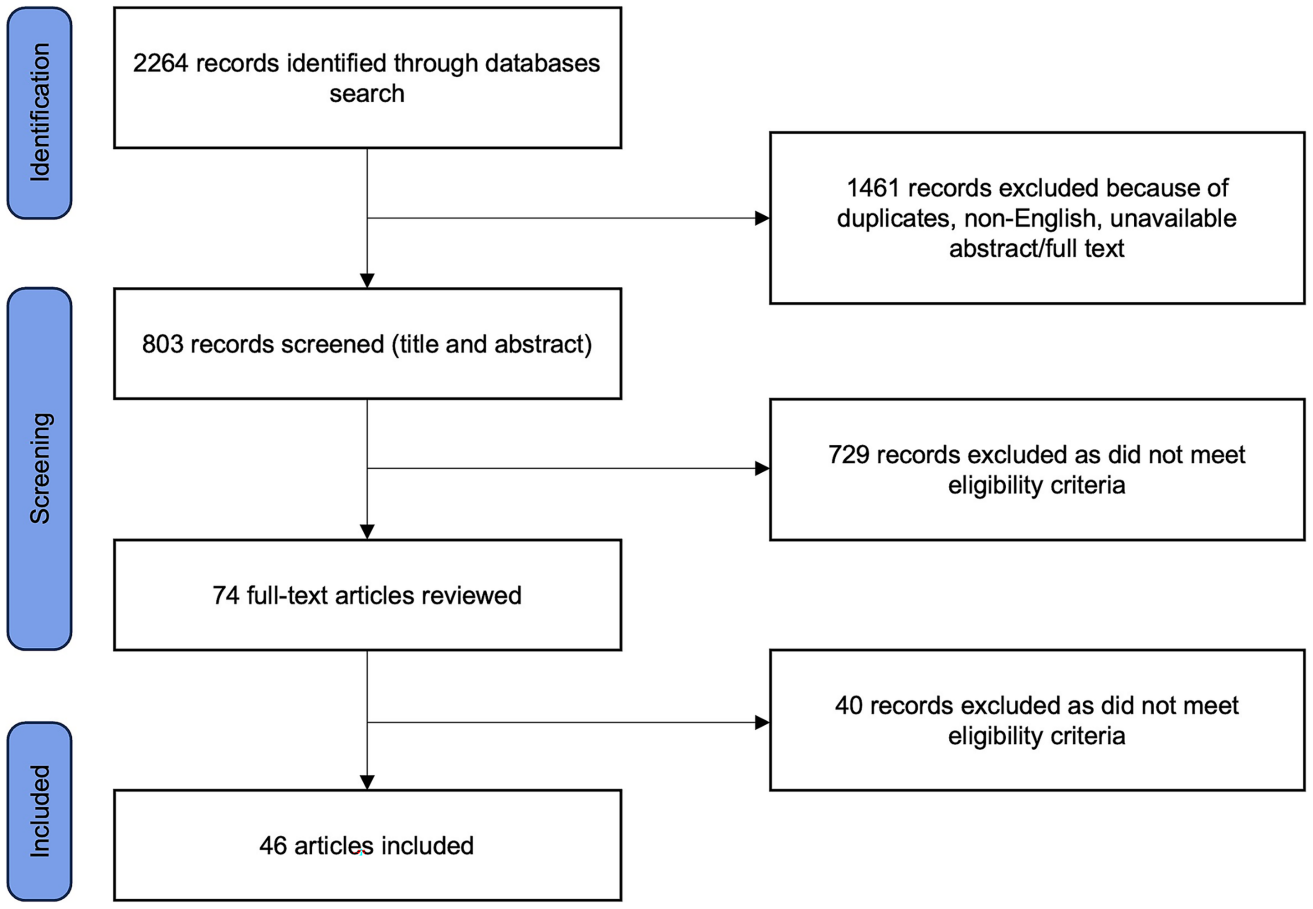
Flowchart of study selection process.

The database search identified a total of 2,264 articles. We first excluded duplicates, as well as studies published in non-English languages or those without available abstract/full text. Titles and abstracts of the remaining 803 studies were reviewed, and 729 more articles were excluded based on inclusion and exclusion criteria. Seventy-four articles underwent full-text review, and a final total of 46 studies were deemed eligible (Figure 1). Among the included articles, 40 studies examined the relationship between anosognosia and NPS in AD, 7 in PD, and 2 in FTD and related syndromes. No studies specifically focused on DLB. Table 1 provides a summary of all included studies, detailing participant characteristics, median age (when available), the domains of NPS investigated, NPS and awareness assessment tools, and key findings. To improve the consistency of reported findings, in this review we grouped NPS into six broad domains that emerged from the included studies and reflect clinical plausibility: (i) depression; (ii) apathy; (iii) affective dysregulation symptoms (i.e., mania, euphoria, pathological laughing, irritability); (iv) impulse dyscontrol symptoms (i.e., disinhibition, agitation, aberrant motor behavior, inattention, tension); (v) psychotic symptoms (i.e., hallucinations, delusions); and (vi) sleep and appetite disturbances. Although this grouping is not based on a universally established consensus, it provides a systematic framework to examine heterogeneous findings across studies. In the included studies, some authors specifically investigated anosognosia with respect to a particular cognitive or behavioral domain (e.g., unawareness of memory loss, unawareness of behavioral disturbances). In these cases, we explicitly report the domain-specific form of anosognosia. Otherwise, the term “anosognosia” is used alone to denote a generalized lack of disease awareness.

**Table 1:** Key findings of identified studies.

Study	Participants (median age, years)	Domains of NPS	NPS assessment	Awareness assessment	Key Findings
Amanzio et al. (83)	29 AD (75)	Apathy impulse dyscontrol	AES DS	AQ-D	Higher apathy and disinhibition in unaware relative to aware subjects.
Castrillo Sanz et al. (4)	127 AD (80)	Global NPS	NPI-Q	CIRS	High NPI-Q score predicts the presence of anosognosia.
Castro et al. (87)	52 PD (60.7)	Global NPS depression	NPI HADS BDI	Subjective complaints	Higher depression and NPS in subjects with subjective cognitive complaints.
Chen et al. (66)	55 AD (76.6)	Depression apathy	CSDD AES	GRAD Subject-informant discrepancy scores	Lower depression in subjects with anosognosia; higher apathy in subjects with anosognosia; higher depression predicts lower anosognosia.
Cines et al. (76)	104 AD (77.5)	Depression	GDS	ARS	Lower depression predicts anosognosia; higher anosognosia predicts lower depression.
Clare et al. (58)	12 AD (71)	Global NPS depression	CAPE HADS	MARS	Positive correlation between anosognosia and global CAPE score; negative correlation between anosognosia and subjects’ depression; positive correlation between anosognosia and caregivers’ depression.
Conde-Sala et al. (52)	164 AD (77.6)	Global NPS depression apathy impulse dyscontrol affective dysregulation appetite	NPI GDS	AQ-D	Higher NPI score in subjects with high AQ-D score relative to low AQ-D score; lower GDS score in subjects with high AQ-D score relative to low AQ-D score; higher apathy, agitation, disinhibition, AMB, irritability, and appetite scores in subjects with high AQ-D score relative to low AQ-D score; in the high AQ-D score group, positive correlation between anosognosia and agitation, disinhibition, AMB, irritability, euphoria, appetite and negative correlation between anosognosia and depression; higher NPI and lower GDS scores predict severe anosognosia.
Derouesné et al. (69)	88 AD (73.2)	Depression apathy	ZD, ZA PBQ	Subject-informant discrepancy scores	Negative correlation between anosognosia and anxiety; positive correlation between anosognosia and apathy.
Gilleen et al. (67)	27 AD (82.4)	Depression	BDI-II	SUMD SAI-E MARS PCRS DEX	Negative correlation between anosognosia and depression.
Horning et al. (77)	107 AD (82.4)	Depression apathy	NRS AES	NRS (Insight)	Higher anosognosia predicts lower depression and anxiety and higher apathy, after controlling for global cognition.
Jacus (63)	20 AD, 20 MCI (80.5, 78.5)	Depression apathy	BDI-II STAI AES	PCRS SCQ	Negative correlation between anosognosia and depression and anxiety; positive correlation between anosognosia and apathy; higher presence of low depression score and high apathy score among subjects with high anosognosia score.
Kashiwa et al. (7)	84 AD (75.5)	Depression impulse dyscontrol	NPI GDS	Subject-informant discrepancy scores	Negative correlation between anosognosia and depression; positive correlation between anosognosia and disinhibition score.
Kelleher et al. (73)	75 MCI (57 AD) (76.4)	Depression	NPI ADS	AD8 discrepancy	Higher depression and anxiety in subjects with preserved insight relative to subjects without preserved insight; higher depression and anxiety predict lower insight.
Lacerda et al. (57)	89 AD (78)	Global NPS depression	NPI CSDD	ASPIDD	Positive correlation between anosognosia and NPI score and depression.
Lehrner et al. (74)	280 SCD (64) 181 naMCI (67) 137 aMCI (70) 43 AD (74) 28 PD (67) 58 PD-naMCI (69) 29 PD-aMCI (69) 211 controls (66)	Depression	BDI	Complaint-performance discrepancy	Negative correlation between anosognosia and depression in aMCI, naMCI, SCD, PD, PD-naMCI, and controls. No significant association in AD and PD-aMCI.
Lopez et al. (78)	181 AD (71.4)	Depression psychosis	Psychiatric assessment	Clinician rating	No association.
Mak et al. (79)	36 AD, 21 MCI (72.6, 69.2)	Depression apathy	GDS AES	AQ-D	Higher apathy predicts anosognosia (AQ-D total and IF only).
Marino et al. (88)	58 PD (not available)	Depression	GDS	PDQ-39 (perceived cognition)	Positive correlation between perceived cognition and depression.
Migliorelli et al. (30)	73 AD (72.9)	Depression affective dysregulation	HAM-D BMS PLACS	AQ-D	Lower dysthymia in subjects with anosognosia; higher mania and pathological laughing score in subjects with anosognosia.
Mikos et al. (91)	37 PD (69)	Apathy	AES	Subject-informant discrepancy scores	Correlation between higher apathy and both greater under- and over-reporting.
Nakaaki et al. (64)	42 AD (71.4)	Depression	Provisional diagnostic criteria for depression in AD	SMQ (discrepancy score)	Higher discrepancy score in subjects without depression relative to subjects with depression.
O’Keeffe et al. (36)	14 FTD, 11 CBD, 10 PSP (not available)	Depression	HADS MET	Composite score for metacognitive awareness, online emergent awareness, online anticipatory awareness	Negative correlation between online anticipatory awareness and depression; positive correlation between metacognitive awareness and empathy.
Oba et al. (65)	118 AD, 47 MCI (79.5, 76.2)	Depression	GDS	Subject-informant discrepancy scores	Lower depression predicts anosognosia.
Orfei et al. (89)	197 PD, 141 MCI-PD, 47 PPD (62.6, 68.6, 73.4)	Depression	BDI	AQ-D	Negative correlation between depression and anosognosia (AQ-D total and IF only).
Reed et al. (81)	57 AD	Depression	Clinician rating	Clinician rating	No association.
Satler and Tomaz (53)	21 AD (78.6)	Global NPS depression	NPI CSDD	AQ-D	Higher NPI score predicts higher anosognosia (AQ-D IF).
Sato et al. (59)	143 AD (72.1)	Global NPS	NPI	AQ-D	Higher NPI score predicts higher anosognosia (AQ-D BEH).
Seltzer et al. (71)	36 AD (74.6)	Depression Affective Dysregulation	CSDD Subject and informant ratings	Subject-informant discrepancy scores	Negative correlation between anosognosia and depression (caregiver rating); positive correlation between anosognosia and irritability (caregiver rating).
Sevush and Leve (72)	128 AD (69.2)	Depression	Subject and informant ratings	Clinician rating	Negative correlation between anosognosia and depression.
Smith et al. (68)	23 AD (75.3)	Depression	GDS	AII	Negative correlation between anosognosia and depression.
Sousa et al. (13)	69 AD (76.8)	Apathy impulse dyscontrol	NPI CSDD	ASPIDD	Positive correlation between anosognosia and apathy and agitation scores.
Spalletta et al. (54)	103 AD, 52 a-MCI, 54 md-MCI (76.3, 71.4, 71.2)	Global NPS apathy impulse dyscontrol affective dysregulation	NPI	AQ-D	Positive correlation between anosognosia (i.e., AQ-D total, AQ-D IF, AQ-D BEH) and NPI score, apathy, agitation, and AMB; positive correlation between anosognosia (i.e., AQ-D total, AQ-D BEH) and irritability.
Starkstein et al. (82)	170 AD (70.5)	Depression apathy affective dysregulation psychosis	HAM-D AS BMS PLACS DPS	AQ-D	Higher psychosis and apathy and lower depression predict anosognosia for cognitive deficits; higher mania and pathological laughing score predict anosognosia for behavioral problems.
Starkstein et al. (90)	33 AD, 33 PDD (70.3, 71)	Depression	HAM-D	AQ-D	Higher depression in PDD relative to AD; higher anosognosia in AD relative to PD.
Starkstein et al. (80)	750 AD (71.6)	Depression impulse dyscontrol	HAM-D DS	AQ-D	Positive correlation between anosognosia and disinhibition.
Starkstein et al. (84)	77 AD (71.5)	Apathy	AS	AQ-D	Anosognosia at baseline predicts higher apathy score at follow-up.
Tondelli et al. (6)	91 EOD, 57 LOD (64.9, 78.6)	Global NPS apathy	NPI	CIRS	Early anosognosia predicts NPI score in all subjects and EOD subgroup; positive correlation between anosognosia and apathy score; anosognosia predicts apathy score in EOD subgroup.
Troisi et al. (70)	42 AD (76.2)	Depression	HAM	HAM-I	Higher depression (psychic subscore, not somatic subscore) in subjects with higher awareness.
Turró-Garriga et al. (85)	177 AD (77.8)	Impulse dyscontrol psychosis	NPI	AQ-D	Disinhibition score predicts persistence of anosognosia between baseline and follow-up; delusion score predicts incidence of anosognosia at follow-up.
van Vliet et al. (75)	142 YOAD, 126 LOAD (61.6, 79.1)	Depression	NPI	GRAD	Negative correlation between anosognosia and depression, more in YOAD than in LOAD.
Verhülsdonk et al. (55)	47 AD (76.5)	Global NPS depression	NPI GDS NOSGER	AQ-D	Positive correlation between anosognosia and NPI score; positive correlation between anosognosia and depression reported by informants (NPI-depression score, NOSGER mood), no correlation between anosognosia and depression reported by subjects.
Vogel et al. (56)	321 AD (76.2)	Global NPS depression	NPI-Q CSDD	ARS MDR	Higher NPI-Q score (total severity score and distress score) in subjects with no insight relative to subjects with full insight classified through ARS; positive correlation between NPI-Q score and MDR score.
Wang et al. (61)	237 MCI (132 A+) (73)	Global NPS apathy impulse dyscontrol affective dysregulation psychosis	NPI	ECog	Higher NPI total score, agitation and disinhibition scores in unaware subjects; earlier onset of NPS (i.e., apathy, agitation, disinhibition, AMB, irritability, delusion, and hallucination) in unaware subjects relative to aware ones.
Yoo et al. (86)	340 PD (67.2)	Global NPS depression	NPI BDI	Complaint-performance discrepancy	Higher NPI total score in subjects with cognitive underestimation; lower depression in subjects with anosognosia.
Yoon et al. (60)	616 early onset AD (62.6)	Global NPS impulse dyscontrol psychosis sleep, appetite	NPI	Clinician rating	Higher NPI score in subjects with anosognosia compared to subjects without anosognosia, only for CDR 0.5–1; higher agitation, AMB, delusion, hallucination, sleep, and appetite scores in subjects with anosognosia.
Zilli and Damasceno, (62)	21 AD (72.4)	Global NPS depression	NPI CSDD	SCQ DIS	No association.

Studies are presented in alphabetical order according to first authors’ names. AD: Alzheimer’s Disease; AD8: Eight-item Informant Interview to Differentiate Aging and Dementia; ADS: Anxiety Depression Scale; AES: Apathy Evaluation Scale; AII: Assessment of Impaired Insight; AMB: Aberrant Motor Behavior; AQ-D: Anosognosia Questionnaire for Dementia (IF: Intellectual Functions; BEH: Behavior); ARS: Anosognosia Rating Scale; AS: Apathy Scale; ASPIDD: Anosognosia Scale for Psychosis and Inhibitory Dysregulation in Dementia; A+: amyloid positive; BDI: Beck Depression Inventory; BDI-II: Beck Depression Inventory – II version; BMS: Bech Mania Scale; CAPE: Behavior Problems Checklist of the Clifton Assessment Procedures for the Elderly; CBD: Corticobasal Degeneration; CIRS: Cumulative Illness Rating Scale; CSDD: Cornell Scale for Depression in Dementia; DEX: Dysexecutive Questionnaire; DIS: Denial of Illness Scale; DPS: Dementia Psychosis Scale; DS: Disinhibition scale; ECog: Everyday Cognition Scale; EOD: Early-Onset Dementia; GDS: Geriatric Depression Scale; GRAD: Guidelines for the Rating of Awareness Deficits; HAM: Hamilton Depression Rating Scale (HAM-D: depression item; HAM-I: insight item); HADS: Hospital Anxiety and Depression Scale; LOAD: Late-Onset Alzheimer’s Disease; LOD: Late-Onset Dementia; MARS: Memory Awareness Rating Scale; MCI: Mild Cognitive Impairment (a-MCI: amnesic MCI; na-MCI: non-amnesic MCI; md-MCI: multidomain-MCI); MDR: Memory Discrepancy Rating; MET: Measure of Empathic Tendency; NPI: Neuropsychiatric Inventory; NPI-Q: Neuropsychiatric Inventory Questionnaire; NPS: Neuropsychiatric symptoms; NOSGER: Nurses’ Observation Scale for Geriatric Patients; NRS: Neurobehavioral Rating Scale; PBQ: Psychobehavioural Questionnaire; PCRS: Patient Competency Rating Scale; PD: Parkinson’s Disease; PDD: Parkinson’s Disease Dementia; PDQ-39: Parkinson’s Disease Questionnaire-39; PLACS: Pathological Laughing and Crying Scale; PSP: Progressive Supranuclear Palsy; SAI-E: Scale for Assessment of Insight-Extended; SCD: Subjective Cognitive Decline; SCQ: Self-Consciousness Questionnaire; SMQ: Short Memory Questionnaire; STAI: State–Trait Anxiety Inventory; SUMD: Scale for the Unawareness of Mental Disorder; YOAD: Young-Onset Alzheimer’s Disease; ZA: Zung Self-Rating Scales for Apathy; ZD: Zung Self-Rating Scales for Depression.

### 3.1 Anosognosia and neuropsychiatric symptoms in Alzheimer’s disease

#### 3.1.1 Global measures of neuropsychiatric symptoms

The literature exploring the relationship between anosognosia and NPS in AD is extensive and relatively well established.

First, a positive correlation between anosognosia—measured either as a continuous variable or as a categorical presence/absence using the AQ-D—and the NPI total score has been consistently reported (52–55). The same association has been observed when anosognosia was assessed using alternative instruments, such as the Anosognosia Rating Scale Memory Discrepancy Rating (4), the Cognitive Insight Rating Scale (56), the Anosognosia Scale for Psychosis and Inhibitory Dysregulation in Dementia (57) and the Memory Awareness Rating Scale (58). Notably, Sato et al. (59) found in a large group of subjects (*n* = 143 AD) that this relationship was specific to anosognosia for behavioral complaints (i.e., AQ-D BEH), whereas anosognosia for cognitive deficits (i.e., AQ-D IF) was not significantly associated with NPS.

In early-onset AD, Yoon et al. (60) reported an association between anosognosia and NPS only in individuals with MCI or mild dementia (Clinical Dementia Rating Scale, CDR = 0,5 or 1), but not in more advanced stages (CDR = 2), suggesting that as dementia progresses, NPS may emerge independently of anosognosia. In addition, Tondelli et al. (6), compared early-onset (EOD) and late-onset dementia (LOD), and found a significant positive correlation between anosognosia and NPS only in EOD. Further supporting this pattern, a longitudinal study in MCI populations showed that individuals with anosognosia had higher NPI global scores at baseline and experienced an earlier onset of NPS over time compared to subjects with preserved awareness (61).

In contrast, Zilli and Damasceno did not observe this association when measuring anosognosia with Self-Consciousness Questionnaire (SCQ) and Denial of Illness Scale (DIS) in a small cohort of subjects (*n* = 21 AD) (62).

#### 3.1.2 Depression

When examining specific neuropsychiatric symptoms, most studies have reported an inverse correlation between anosognosia and depressive symptoms. In other words, individuals who are more aware of their deficits tend to experience more depressive symptoms than those who are unaware (7, 52, 63–72). This relationship has been observed not only in AD dementia but also in MCI and SCD individuals, regardless of amyloid status (73, 74), and has been shown to be stronger in young-onset AD dementia relative to late-onset AD (75). Cines et al. (76) demonstrated that depression can both predict and be predicted by anosognosia in a cross-sectional design on 104 AD individuals. Furthermore, a longitudinal study by Horning et al. (77), found that the levels of awareness at baseline in a sample of 107 AD predicted subsequent development of depressive mood and anxiety, even after controlling for global cognition.

Three of these studies analyzed the concept of depression along with anxiety, which is frequently interpreted as a symptom accompanying depression, and found that increased anosognosia was associated with lower anxiety (63, 73, 77). One study by Derouesné et al. (69) has reported an association between anosognosia with anxiety and not with depression.

It is also important to note that anosognosia can also manifest as lack of awareness of depressive symptoms, a phenomenon referred to as affective anosognosia. For example, two studies reported a positive correlation between anosognosia and depression only when depressive symptoms were assessed by an informant, not by the patient (55, 58). The authors suggested that unaware individuals may appear less depressed simply because they under-recognize and under-report their own depressive symptoms (55).

In contrast, seven studies failed to find an association between anosognosia and depression (53, 56, 62, 78–81). For instance, Lopez et al. (78) relied on psychiatric assessments and clinician ratings rather than standardized questionnaires for measuring NPS and anosognosia. Migliorelli et al. (30) only demonstrated a significant association between increased anosognosia and lower dysthymia, instead of depression.

Only a few studies have reported an inverse association, such that anosognosia was positively correlated with depression (55, 57, 58). More specifically, Lacerda et colleagues (57) showed a positive correlation between anosognosia and both awareness for emotional state and social functioning, whereas two other studies reported this positive correlation only when depression was measured through caregivers (55, 58). Finally, some authors proposed a differential model in which depressive symptoms are negatively related to unawareness for cognitive deficits (i.e., AQ-D IF), but not to unawareness of behavioral symptoms (66, 82). This interpretation underscores the multifaceted nature of anosognosia, suggesting that different domains of unawareness may have distinct relationships with specific neuropsychiatric symptoms.

#### 3.1.3 Apathy

Another significant association is the one between increased levels of anosognosia and greater apathy, observed both in MCI and dementia stages (13, 52, 54, 63, 66, 69, 79, 83, 84). Similarly to depression, apathy appeared to be more strongly associated with anosognosia for cognitive deficits (i.e., AQ-D IF) than with anosognosia for behavioral disturbances (79, 82). Moreover, this association remained significant even after controlling for global cognition (77). Longitudinal studies have also demonstrated that both apathy and anosognosia tend to worsen over time, and that baseline anosognosia predicts an earlier onset and greater severity of apathy at follow-up (61, 84).

When distinguishing between EOD and LOD, including but not limited to AD, the positive association between anosognosia and apathy has been documented only in EOD subjects (6).

#### 3.1.4 Affective dysregulation symptoms

A more limited number of studies have investigated the association between anosognosia and affective dysregulation symptoms, including mania, euphoria, pathological laughing, and irritability. These studies have consistently reported higher scores for these symptoms in individuals with anosognosia compared to those who were aware of their deficits (30, 52, 54, 71, 82), not only in the dementia stages but also in MCI individuals (61). More specifically, Starkstein et al. (82) observed that this association was significant for mania and pathological laughing scores only when considering anosognosia for behavioral disturbances (i.e., AQ-D BEH), rather than anosognosia for cognitive deficits.

#### 3.1.5 Impulse dyscontrol symptoms

Impulse dyscontrol symptoms, including disinhibition, agitation, aberrant motor behavior (AMB), inattention, and tension, have been found to be positively correlated with anosognosia (7, 13, 52, 54, 60, 80, 83, 85), and this association has also been observed in individuals with MCI (61). Moreover, longitudinal evidence has suggested that baseline levels of agitation and AMB may predict the later emergence of anosognosia (54).

#### 3.1.6 Psychotic symptoms

The presence of psychotic symptoms, namely delusions and hallucinations, has also been linked to anosognosia (60, 61, 82, 85). In a longitudinal study, delusional symptoms at baseline were found to predict the persistence of anosognosia at follow-up (85). Conversely, in individuals with MCI, baseline anosognosia was associated with an earlier onset of both delusion and hallucination over time (61). However, one study with a small sample size failed to confirm this association (78).

#### 3.1.7 Sleep and appetite disturbances

Finally, few studies reported an association between higher levels of anosognosia and greater disruption in sleep habits and patterns (60), as well as appetite disturbances (52, 60). These findings suggest that anosognosia may be linked to broader homeostatic and circadian regulatory impairments, although further research is needed to clarify the underlying mechanisms.

### 3.2 Anosognosia and neuropsychiatric symptoms in frontotemporal dementia

Despite being regarded as a core symptom of FTD, particularly its behavioral variant (bvFTD), anosognosia and its association with neuropsychiatric symptoms have been less extensively explored in this form of dementia. O’Koeffe et al. (36) distinguished three different facets of awareness (namely metacognitive, emergent, and anticipatory awareness) in subjects with FTD, Corticobasal Degeneration (CBD) and Progressive Supranuclear Palsy (PSP). They found that FTD individuals exhibited significantly higher levels of anosognosia compared to both other patient groups and controls, and that anticipatory anosognosia (i.e., the inability to predict difficulties in performing a task because one’s own deficits) was negatively correlated with depression while metacognitive awareness (i.e., the declarative knowledge about one’s own abilities) was positively correlated with empathy. The study of Tondelli et al. (6), which investigated differences between EOD and LOD and also included 28 FTD subjects, demonstrated that anosognosia was more frequent in bvFTD, as well as in the behavioral/dysexecutive variant of AD. This study also found that only apathy was positively associated with anosognosia, independent from age and clinical diagnosis. Specifically, higher levels of unawareness at baseline were linked to the subsequent development of apathy.

### 3.3 Anosognosia and neuropsychiatric symptoms in *α*-synucleinopathies

Similar to FTD, the investigation of anosognosia and NPS in α-synucleinopathies remains scarce. A few studies on PD demonstrated that individuals with PD who exhibited greater anosognosia for cognitive difficulties had lower scores on depression scales, consistent with findings in AD (74, 86). In particular, Lehrner et al. (74) showed this association in PD without cognitive impairments and in PD with non-amnesic MCI (not in the small group of PD with amnesic MCI). However, in contrast to AD, higher NPI global scores were observed in individuals with cognitive underestimation (i.e., the tendency to overestimate cognitive difficulties, as opposed to anosognosia) (86). Two additional studies, although not directly measuring anosognosia, reported increased depressive symptoms in PD subjects who expressed greater subjective concerns regarding their cognitive performance (87, 88). Concurrently, Orfei et al. (89) observed a negative association between anosognosia for cognitive impairment (i.e., AQ-D IF) and depression in both PD-MCI and PD without cognitive complaints, though this association was not observed in PDD, despite increasing levels of anosognosia in PD-MCI and PDD, respectively. Starkstein et al. (90) found that, while not directly assessing the relationship between anosognosia and depression in AD and PDD individuals, AD subjects exhibited increased levels of anosognosia and decreased levels of depression, while PDD subjects showed increased levels of depression and decreased levels of anosognosia.

Regarding the relationship with apathy, Mikos et al. (91) documented that PD subjects with inaccurate self-reporting of emotional expressivity—whether over-reporting or under-reporting—had increased levels of apathy, which supports the emotion hypothesis of anosognosia, suggesting that apathy diminished concern about deficits, which in turn leads to inaccurate self-reporting.

No studies have directly examined the association between anosognosia and NPS specifically in DLB. However, it is worth noting that some of the articles discussed in Section 3.1 included individuals with MCI, particularly multi-domain MCI, regardless of amyloid status. Therefore, potential diagnoses other than AD, such as DLB, may be relevant in those cohorts. Specifically, these studies confirmed increased levels of depression and anxiety in individuals with decreased anosognosia (73) and higher NPI total score, agitation, and disinhibition in subjects with greater anosognosia (61). Additionally, Tondelli et al. (6) reported a positive correlation between anosognosia and apathy score in a cohort of EOD, including 12 individuals with DLB.

## 4 Discussion

**Figure 2:**
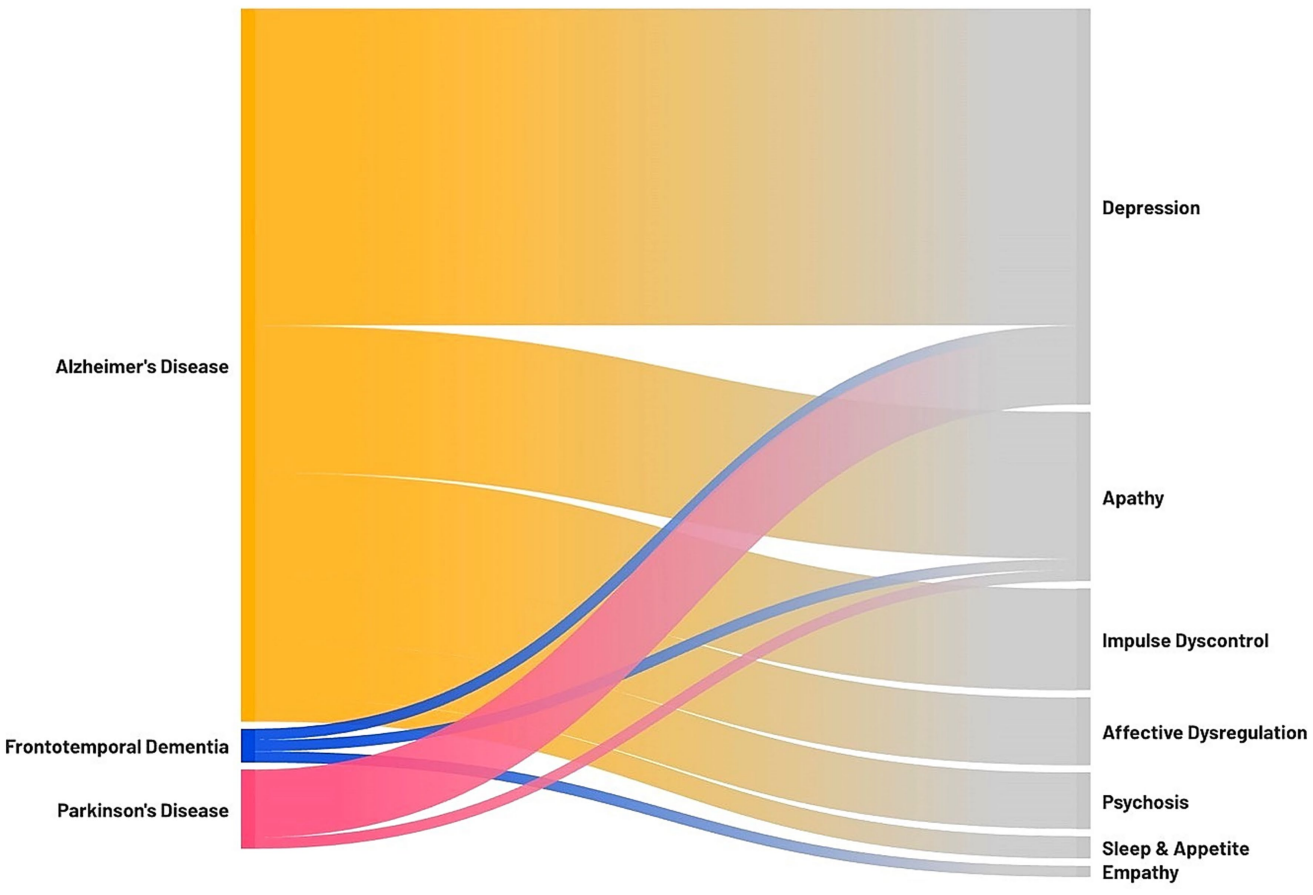
Sankey diagram illustrating the association between anosognosia and neuropsychiatric symptoms in neurodegenerative dementias. On the left, the neurodegenerative diseases investigated in the review with significant findings; on the right, the neuropsychiatric symptoms associated with anosognosia. The thickness of each connection represents the number of studies focusing on a specific association (i.e., the association between anosognosia and depression was assessed in 28 studies on AD, 1 study on FTD, and 6 studies on PD; the association between anosognosia and apathy was assessed in 13 studies on AD, 1 study on FTD, and 1 study on PD; the association between anosognosia and impulse dyscontrol symptoms was assessed in 9 studies on AD; the association between anosognosia and affective dysregulation symptoms was assessed in 6 studies on AD; the association between anosognosia and psychosis was assessed in 5 studies on AD; the association between anosognosia and sleep and appetite was assessed in 2 studies on AD; the association between anosognosia and empathy was assessed in 1 study on FTD).

This narrative review provides an overview of the current evidence on the association between anosognosia and NPS, two common and critical symptoms in neurodegenerative dementias, which substantially impact patient management and quality of life. The findings highlight a complex interplay between these two clinical features, with significant variability depending mostly on the specific domains of anosognosia and NPS under investigation, as well as the neurodegenerative condition examined. The Sankey diagram reported in Figure 2 summarizes the findings of this review, illustrating which associations between NPS and anosognosia have been investigated in each neurodegenerative disease.

The most substantial body of literature concerns AD, where anosognosia was initially characterized and has since been the subject of extensive investigation. In AD, a consistent positive association has been observed between anosognosia and global scores of NPS, pointing to a potential causal link and a shared neurobiological substrate underlying their co-occurrence (54, 61). Conversely, in FTD, studies exploring the relationship between anosognosia and NPS remain scarce and are even absent in the linguistic variants of FTD. The existing articles suggest a higher prevalence of NPS in unaware subjects (6, 36), but their limited number and the heterogeneity of the included patient populations prevent definitive conclusions. Importantly, it could also be debated whether the lack of awareness of behavioral symptoms in FTD represents true anosognosia or rather stems from the loss of social manner and decorum that characterize these patients, as part of a broader disinhibition syndrome. In other words, individuals with FTD may appear unaware of their behavior, yet they might be indifferent to it because of the absence of recognition regarding the social inappropriateness of their actions. Similarly, evidence regarding *α*-synucleinopathies remains scarce and inconclusive. Two studies report, in contrast to AD, higher NPS in PD individuals with cognitive underestimation or subjective complaints (86, 87), while in DLB dedicated studies focusing exclusively on the association between anosognosia and NPS are lacking.

Notably, more informative findings arise from studies that analyze specific NPS domains individually, with particular emphasis on depression and apathy. In AD, the relationship between anosognosia and depression appears to be robustly inverse, with greater awareness of cognitive decline associated with increased depressive symptoms (7, 52, 65, 66, 77, 82). Despite more limited evidence, the same association is suggested in FTD (36) and PD individuals (74, 86–90). Two main hypotheses have been proposed to explain this association. One posits that a depressed mood—characterized by a gloomy perspective on the future and pessimistic ideation about current condition, may lead to a cognitive negative bias, resulting in underestimation of one’s abilities (92). The alternative hypothesis suggests that reduced awareness of one’s deficits may act as a psychological defense mechanism, protecting individuals from emotional distress (92). In essence, the first hypothesis frames depression as a determinant of increased awareness, while the second conceptualizes anosognosia as a protective factor against depression. Interestingly, some studies have reported a negative association between anosognosia and dysthymia, but not with major depressive disorder. This finding has led to the suggestion that major depression may reflect a more biologically driven mood disorder, whereas dysthymia could represent a more reactive emotional response to cognitive decline – particularly in individuals with preserved awareness (71). Additionally, the possibility of *affective anosognosia*, or a lack of awareness regarding one’s own depressive symptoms, must be considered. In such cases, patients may underreport symptoms, contributing to discrepancies in the observed associations (31). Finally, anosognosia is increasingly recognized as a multidimensional construct, with distinct domains potentially relating differently to NPS. For example, Starkstein et al. (58) proposed that only unawareness of cognitive deficits is associated with depressive symptoms, suggesting that domain-specific distinctions in anosognosia may clarify inconsistent findings. Moreover, empirical evidence indicates that different dimensions of anosognosia are not necessarily interrelated. For instance, unawareness of having a disease has been shown to correlate with anosognosia for apathy, but not with unawareness of depressive symptoms (72). These nuances underscore the need for a more refined conceptual and methodological approach in future research.

In the domain of apathy, anosognosia has been consistently associated with increased apathy severity in both AD (52, 54, 61, 69, 77, 83, 84) and FTD (6). In regard to PD individuals, the association with increased apathy has been shown both for over- and under-reporting of emotional expressivity, suggesting that apathy determines a general reduced concern about one’s own deficits (91). To explain this association, it has been proposed that apathy may contribute to anosognosia through mechanisms such as affective blunting and diminished performance monitoring, whereby cognitive errors lose emotional salience and fail to trigger self-awareness processes (92, 93). Conversely, from a metacognitive perspective, anosognosia may exacerbate apathy by impairing self-reflection and reducing the capacity for goal-directed behavior and motivation, processes that are reliant on intact metacognitive functioning (94). Starkstein et al. (61) further proposed a longitudinal model in which anosognosia and apathy represent distinct but sequential manifestations of progressive frontal lobe dysfunction. According to this framework, anosognosia may emerge early as an initial response to frontal damage, while apathy may develop later as frontal degeneration becomes more widespread. Interestingly, both apathy and anosognosia have been associated to frontal regions: apathy has been related to dorsal lateral and medial prefrontal cortex, orbitofrontal cortex, and anterior cingulate cortex (ACC) (95, 96), while ACC has been involved in the error monitoring processes, which are fundamental for the recognition of one’s cognitive deficits. Thus, ACC dysfunction could be a shared biological hallmark of both apathy and anosognosia (92). Notably, discrepancies in the severity and impact of apathy have been reported across patient subgroups, such as in comparisons between EOD and LOD. Some studies suggest that EOD may be associated with more pronounced apathy, potentially due to the disruption of work, family, and social roles, which may heighten demotivation and disengagement (3).

The literature on other NPS, including affective dysregulation, impulse dyscontrol disorders, and psychosis, is more sparse. However, available evidence suggests a positive association with anosognosia. In particular, unawareness of behavioral symptoms may be a key factor in the emergence of affective dysregulation symptoms (82). The relationship between anosognosia and impulse dyscontrol symptoms supports the involvement of frontal circuits in both phenomena, since frontal regions such as the ACC and orbitofrontal cortex (OFC) and their connections have been implicated in agitation, AMB, and disinhibition (7, 97, 98) as well as in anosognosia (99) suggesting a common neuroanatomical basis to explain their co-occurrence. Additionally, reduced connectivity in the Default Mode Network (DMN), a brain network involved in self-referential processing, has been associated with both anosognosia (100) and NPS (101), particularly hyperactivity symptoms. A recent review further points to the amygdala and limbic system as crucial hubs in this network, given their role in emotional regulation and behavioral control. The amygdala appears highly vulnerable to neurodegeneration, and its disconnection from OFC and frontal cortices may contribute to disinhibition, impulsivity, and agitation. These converging results suggest a crucial role for the disruption of interconnected frontal and limbic circuits and their interactions with the DMN (102). Finally, a bidirectional relationship between anosognosia and psychosis has been shown, suggesting that deficits in reality monitoring – a cognitive function essential for distinguishing internally generated thoughts from external realit—may underlie both anosognosia and psychotic symptoms (60).

Some limitations should be highlighted and may explain part of the inconsistency across studies. First, there is considerable methodological variability in the assessment of both anosognosia and NPS, particularly in relation to depressive symptoms. Anosognosia extends beyond cognitive deficits to include unawareness for behavioral disturbances, personality changes, and deficits in social cognition. However, the operationalization of this construct is not homogeneous, and appropriate standardized instruments are still needed to capture this complexity. Similarly, the tools used to assess NPS vary widely across studies, contributing to inconsistencies in reported associations. A second limitation concerns sample heterogeneity, including small sample sizes, differences in patient selection, and variability in disease severity (e.g., MCI versus dementia). In particular, many studies on AD relied on clinical criteria without biomarker confirmation, or MCI cohorts without verified underlying AD pathology, thereby introducing additional variability. Finally, the included studies display substantial heterogeneity in design, diagnostic criteria, measurement tools, and sample characteristics. This variability was not systematically controlled for or coded through a formal quality assessment framework (e.g., risk of bias/quality chart), given the narrative design of the present review. This further constrains the interpretability of pooled findings and underscores the need for standardized methodological approaches in future work.

## 5 Conclusion

This narrative review highlights the complex interplay between anosognosia and NPS across neurodegenerative dementias. While the association with global NPS, depression, and apathy in AD is well-documented, evidence in FTD and *α*-synucleinopathies, as well as data regarding impulse dyscontrol symptoms, affective dysregulation, and psychosis remains limited and inconclusive. Patient selection criteria and methodological heterogeneity in assessing both anosognosia and NPS represent a major challenge, contributing to variability in findings. Standardized and multidimensional approaches, which extend beyond anosognosia for cognitive deficits to include behavioral and social domains, are needed to clarify the underlying neurobiological mechanisms linking anosognosia to NPS. A better understanding of this relationship is critically needed as it has potential implications for early diagnosis, personalized intervention strategies, and may help improve both patients’ and caregivers’ quality of life in neurodegenerative dementias.
